# Effects of exercise on neuromuscular junction components across age: systematic review of animal experimental studies

**DOI:** 10.1186/s13104-015-1644-4

**Published:** 2015-11-24

**Authors:** Walter Krause Neto, Adriano Polican Ciena, Carlos Alberto Anaruma, Romeu Rodrigues de Souza, Eliane Florencio Gama

**Affiliations:** Laboratory of Morphoquantitative Studies and Immunohistochemistry, Physical Education Department, São Judas Tadeu University, Unidade Mooca, Rua Taquari, 546, Mooca, P.O Box: 03166-000, São Paulo, SP Brazil; Laboratory of Histology and Electron Microscopy, Physical Education Department, “Julio de Mesquita Filho” São Paulo State University, Rio Claro, SP Brazil

**Keywords:** Myoneural junction, Strength training, Aerobic training, Myofibers

## Abstract

**Background:**

During almost one-third of our life, maturation of the nervous system promotes strength and muscle mass increase. However, as age advances, the nervous system begins to suffer a slow and continue reduction of its functions. Neuromuscular junction (NMJ) is one of the structures of which change due to aging process. Physical training leads to significant adjustments in NMJs of young and aged animals. Nevertheless, studies that aimed to investigate this effect have, in many cases, methodological variables that may have some influence on the result. Thus, this study aimed to carry out a systematic review about the effects of exercise training on the NMJ compartments of young, adult and aged animals.

**Results:**

We searched PubMed, Google Scholar, Science Direct, Scielo and Lilacs databases for animal experimental studies that studied exercise effects on the NMJs components across age. After inclusion and exclusion criteria, we included nine articles in systematic review and two for meta-analysis (young/adult NMJ).

**Conclusions:**

We identified that exercise training cause NMJ hypertrophy on young animals and NMJ compression on aged ones. However, many methodological issues such as age, skeletal muscle and fibers type, and type of exercise and training protocol might influence the results.

**Graphical abstract:**
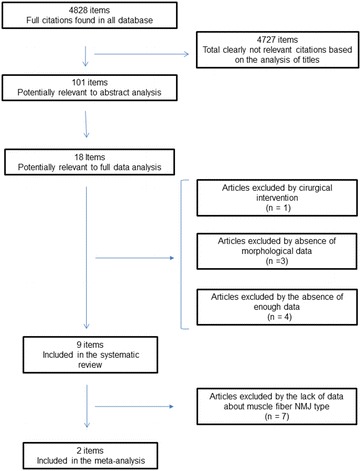
Flow gram is actually to be show at results section as Fig 1

## Background

The neuromuscular junction (NMJ) is a synapse site from peripheral nervous system that allows communication between α-motoneuron and skeletal muscle fibers [1]. NMJ architecture is formed by pre- and post-synaptic compartments. Each compartment consists of several components, such as peripheral axon, myelin sheath, Schwann cells, acetylcholine (ACh) vesicles and receptors (AChR), acetylcholinesterase enzyme, and muscle basal lamina.

One classical feature of this morphological structure is the great plasticity that it suffers across age [2]. Experimental data demonstrated greater NMJ density in juvenile than adult rats [3]. From young to adult age, skeletal muscle fiber goes from a multi-innervated condition to a unique NMJ/muscle fiber state. As time passes by, nervous system begins to suffer a slow and continued reduction of its functions [4]. At late ages, NMJ begins to undergo a process of functional denervation, leading to compensatory functional hypertrophy frame [5–7]. This mechanism is an attempt to prevent the muscle fibers are permanently denervated and undergo apoptosis. This process may lead skeletal muscle to sarcopenia process.

Many strategies are studied in order to stop and/or reverse the sarcopenia process and muscle strength decrease resulted from the advanced biological age [8]. Undoubtedly, one of the most researched and used strategy is physical exercise [9]. Physical exercise can be considered as any physical activity previously scheduled. The relationship between physical exercise and NMJ is studied since the middle of last century. Exercise can promote positive changes in NMJ and thereby promote functional capacity improvement of young and old human and animals [10]. Therefore, this type of intervention is extremely important to maintain the physical functional condition over time. Despite some information on the effect of exercise training on the structure of the NMJ, many studies analyzed different lab techniques, skeletal muscles, exercise types and training protocols [11–14]. These methodological differences might lead us to conflicting, confusing and/or divergent results.

Many molecules have been proposed to participate at NMJ adaptation to exercise [10]. Recently, Nishimune and colleagues [10] reviewed literature-showing probability of molecules such as *insulin like growth factors* (IGFs), *Bassoon* protein, neurotrophin 4 (NT-4) and other to induce NMJ physiological and morphological adaptation to training. However, more data are needed and further mechanisms might be involved in this scene.

Currently, the clinical literature uses systematic reviews and meta-analysis to identify possible studies methodological differences, quality of the surveyed studies on a given topic and the most suitable intervention for a specific treatment. Therefore, this study aimed to carry out a systematic review on the effects of exercise on NMJ compartments of young, adult and aged animals.

## Results

### Studies selected

**Fig. 1:**
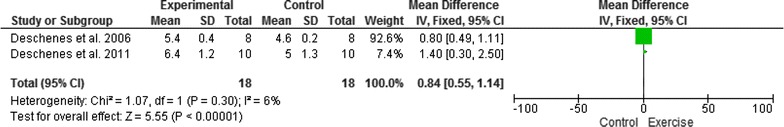
Forest plot presented information about endurance training effects on nerve terminal branch number of young and adult slow-twitch NMJ

After initial intersection of *mesh terms*, *entry terms* and/or related keywords, the search identified 4828 articles titles. From this point, two independent evaluators (WKN and EFG) read all titles. Next, abstracts were selected using PRISMA [15] suggestion (as mentioned in “Methods” section). From all, 101 articles were used for initial abstract analysis. Abstracts should contain sufficient data on parameters of the NMJ components, animal studied and treatments and/or interventions used in the studies. From this, 18 articles were included for full text analysis. After inclusion and exclusion criteria, nine papers were included to systematic review and two for meta-analysis (young/adult only). The selection process is shown in Fig. 1.

Regarding the articles about cellular and molecular mechanisms, we discussed search results directly in the “Discussion” section.

### Animal strain

Within the selected articles, the following strains of rodents have been used: Sprague–Dawley rats [12, 13, 16, 17], Fisher 344 rats [18, 19], Wistar rats [20] and C57BL/6NNia mice [21, 22].

### Animal age and gender

All papers presented data about exercise training effect on NMJs components of young or adult animals. Of those, four papers also studied the exercise effect on aging NMJs parameters [18, 19, 21, 22]. All studies used male animals.

### Type of exercise interventions

Most exercise interventions were endurance [1, 12, 16–18, 20, 22]. Only two papers intervened through resistance training [13, 19].

### Training parameters

Endurance training duration varied from 6 to 15 weeks and 5×/week frequency. Resistance Training studies duration took 7 weeks and 3×/week. Neither article did testing session to prescribe exercise intensity. Training information are shown in the Table 1.

**Table 1:** Description of training parameters from each article included for systematic review

Reference	Training type	Duration (weeks)	Frequence (times/week)	Protocol
Crockett et al. [16]	Endurance	15	5	Intensity was increased during the first 10 weeks, reaching 26.8 m/min, 15 % grade, for 30 min, 3×/week over the final 5 weeks. In alternating days, the animals sprinted 30 s at 42.9 m/min with 30 s interval at 26.8 m/min for 35 min total. All sessions had a 5 min warm-up and ended with 20 min at 26.8 m/min
Andonian and Fahim [21]	Endurance	8	5	Training started with walk-jog pace of 15–20 m/min for 30 min. As the animals became familiar with the equipment, it gradually increased intensity for 25–28 m/min for 60 min per day. This intensity was maintained for the final 4 weeks of training
Waerhaug et al. [20]	Endurance	6	5	All animals trained as close to exhaustion as possible. The speed was gradually increased and progressed from 6 m/min for 8–12 min at the onset of training, at 15 m/min for 20–24 m/min midway in the period and at 22 m/min for 30–35 min during the last week
Deschenes et al. [12]	Endurance	12	5	High-intensity: Animals ran 24 m/min, during 20 min per session, with gradually grade increases from 0 to 25 % inclination. Low intensity: Animals ran 24 m/min, 0 % grade, with duration gradually increased from 20 to 90 min per training session
Fahim [22]	Endurance	12	5	Exercise started at moderate walk-jog pace of 15–20 m/min for 30 min. As the mice became familiar, velocity and duration gradually were increased to 25–28 m/min for 60 min/day
Deschenes et al. [13]	Resistance	7	3	Training consisted of Ladder climbing movements. Each animal climbed the ladder for 10 times, separated by 2 min of rest interval. Load was gradually increased from 50 to 535 g over the weeks
Deschenes et al. [17]	Endurance	10	5	Animals trained for a period from 20 to 60 min with pace held at 25 m/min and 0 % grade inclination
Deschenes et al. [18]	Endurance	10	5	Exercise started with 15 min duration at speed of 7.5 m/min at 0 % grade. Over the weeks, duration and speed were gradually increased to 60 min and 15 m/min, respectively
Deschenes et al. [19]	Resistance	7	3	Ladder training consisted of eight climbs/session, initially with 50 % of body mass with 30 g increments added weekly

### Exercise effect on the NMJs components

#### Young and adult NMJ

##### Pre-synaptic compartment

Most studies used Soleus muscle and showed that endurance training increased nerve terminal, total area, nerve terminal length, and branching complexity [12, 16, 18, 20, 21]. However, these changes might be different depending on the muscle type function and muscle fiber quality [18]. Three studies analyzed different muscles such as EDL [20, 21], Gluteus maximus [22] and Plantaris [18]. One article showed similar results between Soleus and EDL muscles [21], however, another study failed to demonstrate it [20]. One article demonstrated that NMJ adapted differently depending on the muscle fiber type [18].

Two papers analyzed Soleus and Plantaris muscles though resistance training [13, 19]. Neither studies presented any significant changes at this compartment. Data are shown in Table 2.

**Table 2:** Data description regarding exercise type, muscle analyzed, and primary results for young and adult NMJ pre- and post-synaptic compartment

Reference	Exercise type	Muscle	Pre-synaptic compartment	Post-synaptic compartment
Crockett et al. [16]	Endurance	Soleus and vastus lateralis	Not measured	Vastus lateralis = ↑ end plate cholinesterase activity (fast twitch myofiber)
Andonian and Fahim [21]	Endurance	Soleus and EDL	↑ Nerve terminals (both muscles)	Not measured
Waerhaug et al. [20]	Endurance	Soleus and EDL	↑ Nerve terminal area and length (soleus)	Not measured
Deschenes et al. [12]	Endurance	Soleus	↑ Total area (both intensities)	↑ Total area (both intensities)
Fahim [22]	Endurance	Gluteus maximus	↑ Nerve terminal area	Not measured
Deschenes et al. [13]	Resistance	Soleus	High pre to post correlation	↑ Total area and perimeter
Deschenes et al. [17]	Endurance	Soleus	↑ Total Length of branching and branching complexity (both myofiber types)	↓ Pre to post coupling (both myofiber types)
Deschenes et al. [18]	Endurance	Soleus and plantaris	Soleus Slow-twitch = ↑branch number, total branch length and branching complexity; Fast-twitch = not changed; Plantaris Slow-twitch = ↓branching complexity; Fast-twitch = not changed	Soleus Slow-twitch = ↑total perimeter, area and stained area; Fast-twitch = not changed; Plantaris Slow-twitch = ↓total and stained perimeter and total and stained area; Fast-twitch = not changed
Deschenes et al. [19]	Resistance	Soleus and plantaris	Soleus Slow-and fast-twitch = not changed; Plantaris Slow-and fast-twitch = not changed;	Soleus Slow-twitch = ↑End plate dispersion; Fast-twitch = ↑Total and stained areas; Plantaris Slow-and fast-twitch = Not changed;

##### Post-synaptic compartment

One article showed endplate cholinesterase activity increase in the vastus lateralis muscle [16]. Two studies demonstrated total area and perimeter increase at both endurance and resistance trained rodents [12, 13]. One study showed pre-to-post-synaptic coupling decrease in both myofiber types of soleus muscle after endurance training [17]. Two papers visualized specific NMJ adaptation per muscle fiber types [18, 19]. Endurance training increased total perimeter and area of slow-twitch myofibers of the soleus muscle, despite these components are decreased in fast-twitch ones [18]. By other side, resistance training increased endplate dispersion of slow-twitch and total area of fast-twitch myofibers of soleus muscle [19].

#### Aged NMJ

##### Pre-synaptic compartment

Three papers analyzed the endurance training effect on aged soleus [18, 21], EDL [21], gluteus maximus [22] and plantaris muscles [18]. In general, endurance training is shown to decrease perimeter and branch numbers of soleus and EDL [21] and decrease terminal area of gluteus maximus [22]. By fiber type analysis, endurance training decreased average branch lengths and branch length and complexity of slow-twitch myofibers of soleus and plantaris, respectively. In addition, resistance training appeared not to change any pre-synaptic component of aging NMJs [19].

##### Post-synaptic compartment

Two papers investigated the post-synaptic compartment of aged soleus and plantaris myofiber types [18, 19]. Endurance training reduced stained perimeter and increased pre-to-post-synaptic coupling of soleus and plantaris slow-twitch myofibers, respectively [18]. However, fast-twitch myofibers appeared not to be change by training [18, 19]. Surprisingly, resistance training only affected fast-twitch myofibers of soleus muscle by increase total area and decrease endplate dispersion. Data are presented in the Table 3.

**Table 3:** Data description about exercise type, muscles analyzed and primary results for aged NMJ pre- and post-synaptic compartment

Reference	Exercise type	Muscle	Pre-synaptic compartment	Post-synaptic compartment
Andonian and Fahim [21]	Endurance	Soleus and EDL	↓ Perimeter and branch numbers (soleus)	Not measured
Fahim [22]	Endurance	Gluteus maximus	↓ Nerve terminal area	Not measured
Deschenes et al. [18]	Endurance	Soleus and plantaris	Soleus Slow-twitch = ↓average branch length; Fast-twitch = ↑branch number; Plantaris Slow-twitch = ↓branch number, total branch length and branching complexity; Fast-twitch = not changed	Soleus Slow-twitch = ↓stained perimeter; Fast-twitch = not changed Plantaris Slow-twitch = ↑pre to post coupling; Fast-twitch = not changed
Deschenes et al. [19]	Resistance	Soleus and plantaris	Soleus Slow-twitch = not changed Fast-twitch = not changed Plantaris Slow-twitch = not changed Fast-twitch = not changed	Soleus Slow-twitch = not changed Fast-twitch = ↑total and stained area; ↓end plate dispersion; Plantaris Slow-twitch = not changed Fast-twitch = not changed

### Meta-analysis

Meta-analysis was apply for slow and fast-twitch NMJs nerve terminals and post-synaptic endplate structures. For nerve terminal analysis, we included branch number, total branch length (µm), average branch length (µm), branching complexity and pre-to post-synaptic coupling. Further, we analyzed post-synaptic endplates and included total and stained endplate perimeter (µm), total and stained endplate area (µm^2^) and Endplate dispersion (%).

### Data synthesis

All data are presented by the analysis of two articles and 36 animals included [17, 18]. Only endurance training of young and adult rodent data was considered. Age effect was not done at this point because included articles did not present enough internal statistical data. Resistance training articles did not present similar NMJ measurement techniques. This fact turned impossible to meta-analysis evidence from resistance-trained studies.

### Slow-twitch nerve terminals

#### Branch number

Forest plot analysis demonstrated homogeneity (p = 0.30, I^2^ = 6 %) between studies and endurance exercise appeared to increase nerve terminal branch number [p < 0.00001, 95 % CI, 0.84 µm (0.55–1.14)]. Figure 1 presented data and forest plot.

### Total branch length

**Fig. 2:**
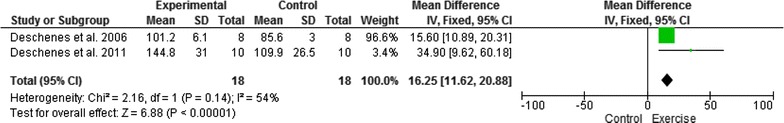
Forest plot presented information about endurance training effects on nerve terminal total branch length of young and adult slow-twitch NMJ

Data showed moderate to high heterogeneity level (p = 0.14, I^2^ = 54 %) between studies. Nevertheless, training period increased total branch length on nerve terminals [p < 0.00001, 95 % CI, 16.25 µm (11.62–20.88)]. Figure 2 presented data and forest plot.

### Average branch length

**Fig. 3:**
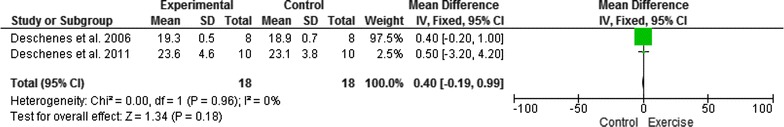
Forest plot presented information about exercise effects on average branch length of the nerve terminals of young and adult slow-twitch NMJ

Presented analysis demonstrated high homogeneity (p = 0.96, I^2^ = 0 %) between studies. Endurance training did not affect average branch length (p = 0.18). Data are presented in Fig. 3.

### Branching complexity

**Fig. 4:**
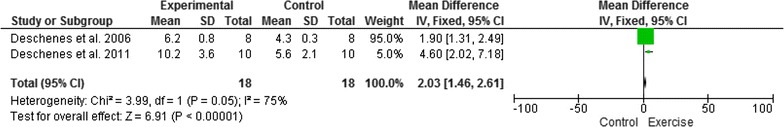
Forest plot presented information about exercise effects on branching complexity of the nerve terminals of young and adult slow-twitch NMJ

Data showed moderate to high heterogeneity between studies (p = 0.05, I^2^ = 75 %). Exercise training appeared to increase nerve terminals branching complexity [p = 0.00001, 95 % CI, 2.03 (1.46–2.61)]. Data are presented in Fig. 4.

### Slow twitch endplate

#### Total perimeter

**Fig. 5:**
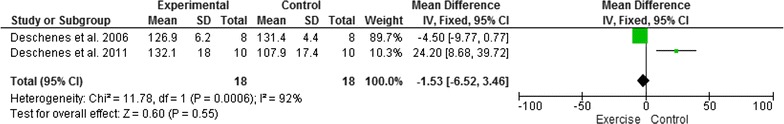
Forest plot presented information about exercise effects on end plate total perimeter of young and adult slow-twitch NMJ

Forest plot data demonstrated high heterogeneity level (p = 0.0006, I^2^ = 92 %). Overall effect test showed that endurance training did not affect endplate total perimeter (p = 0.55). Data are presented in Fig. 5.

### Stained perimeter

**Fig. 6:**
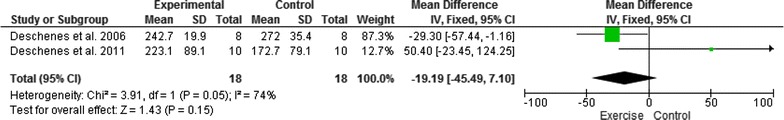
Forest plot presented information about exercise effects on end plate stained perimeter of young and adult slow-twitch NMJ

Data showed high heterogeneity level (p = 0.05, I^2^ = 74 %) between studies. Test for overall effect did not demonstrate any change (p = 0.15). However, coefficient interval showed to be quite wide [95 % CI, −19.19 µm^2^ (−45.49 to 7.10)]. Forest plot and data are showed in Fig. 6.

### Total area

**Fig. 7:**
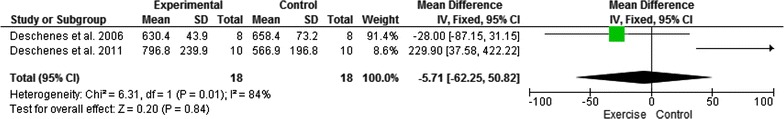
Forest plot presented information about exercise effects on end plate total area of young and adult slow-twitch NMJ

Forest plot demonstrated high level of heterogeneity between studies (p = 0.01, I^2^ = 84 %). Coefficient interval demonstrated wide range effect [95 % CI, −5.71 µm^2^ (−62.25 to 50.82)]. Overall effect did not demonstrate any changes (p = 0.84) (Fig. 7).

### Stained total area

**Fig. 8:**
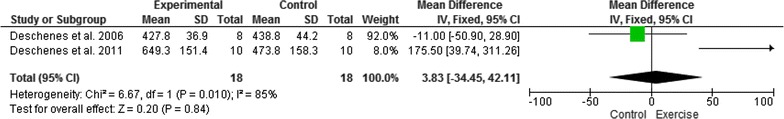
Forest plot presented information about exercise effects on total end plate stained area of young and adult slow-twitch NMJ

Forest plot showed high heterogeneity level (p = 0.01, I^2^ = 85 %) between studies. Test for overall effect did not show any training effect (p = 0.84), although demonstrated a great coefficient interval [95 % CI, 3.83 µm^2^ (−34.45, 42.11)]. Data are showed in Fig. 8.

### Dispersion

**Fig. 9:**
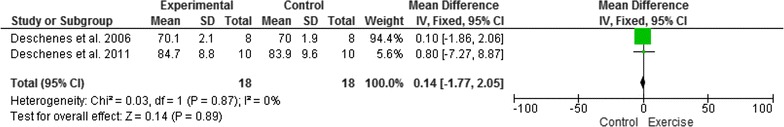
Forest plot presented information about exercise effects on end plate dispersion of young and adult slow-twitch NMJ

Data presented homogeneity (p = 0.87, I^2^ = 0 %) between studies. Endurance training did not affect endplate dispersion (p = 0.89). Figure 9 presents data and forest plot.

### Pre-to post-synaptic coupling

**Fig. 10:**
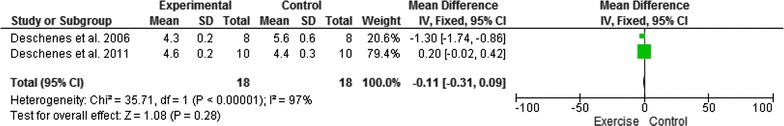
Forest plot presented information about exercise effects on end plate pre- to post-synaptic coupling of young and adult slow-twitch NMJ

Forest plot demonstrated high heterogeneity level (p < 0.00001, I^2^ = 97 %) between studies. Exercise training appeared not to affect pre- to post-synaptic coupling (p = 0.28) (Fig. 10).

### Fast-twitch nerve terminals

#### Branch number

**Fig. 11:**
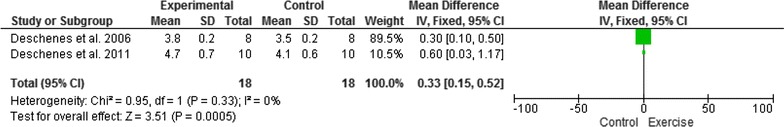
Forest plot presented information about exercise effects on fast-twitch nerve terminal branch number

Overall effect testing demonstrated that endurance training increased branch number of fast twitch nerve terminals [p = 0.0005, 95 % CI, 0.33 µm (0.15, 0.52)]. Data are presented in Fig. 11.

#### Total branch length

**Fig. 12:**
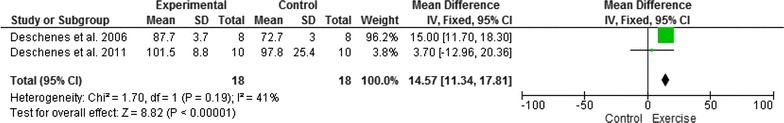
Forest plot presented information about exercise effects on fast-twitch terminal nerve total branch length

Forest plot showed moderate heterogeneity level (p = 0.19, I^2^ = 41 %). Endurance training appeared to increase total branch length [p < 0.00001, 95 % CI, 14.57 µm (11.34, 17.81)]. Data are presented in Fig. 12.

#### Average branch length

**Fig. 13:**
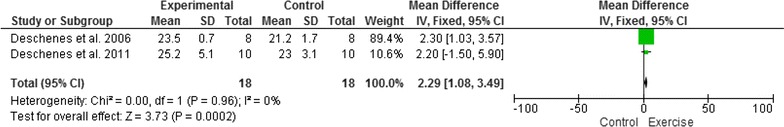
Forest plot presented information about exercise effects on fast-twitch terminal nerve average branch length

Data demonstrated that endurance training appeared to increase average branch length by 2.29 µm [p = 0.0002, 95 % CI (1.08, 3.49)]. Data and forest plot are presented in Fig. 13.

#### Branching complexity

**Fig. 14:**
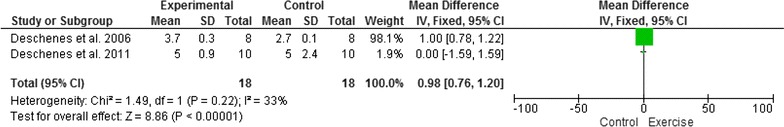
Forest plot presented information about exercise effects on fast-twitch nerve terminal branching complexity

Test for overall effect showed that endurance training appeared to increase branching complexity [p < 0.00001, 95 % CI, 0.98 µm (0.76, 1.20)]. Forest plot and data are presented in Fig. 14.

### Fast-twitch endplate

#### Total perimeter

**Fig. 15:**
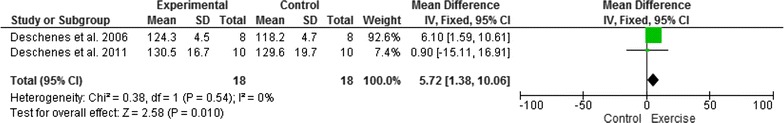
Forest plot presented information about exercise effects on fast-twitch end plate total perimeter

Data demonstrated that endurance training appeared to increase total perimeter by 5.72 µm^2^ [p = 0.01, 95 % CI (1.38, 10.06)]. Data are showed in Fig. 15.

#### Stained perimeter

**Fig. 16:**
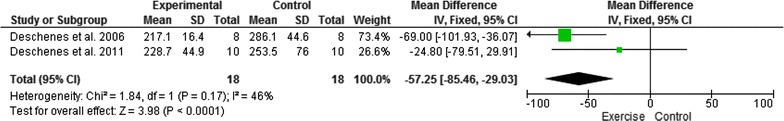
Forest plot presented information about exercise effects on fast-twitch end plate stained perimeter

Forest plot demonstrated moderate heterogeneity level (p = 0.17, I^2^ = 46 %) between studies. Endurance training appeared to affect stained perimeter by reducing it 57.25 µm^2^ [p < 0.0001, 95 % CI (−85.46, −29.03)]. Data and forest plot are presented in Fig. 16.

#### Total area

**Fig. 17:**
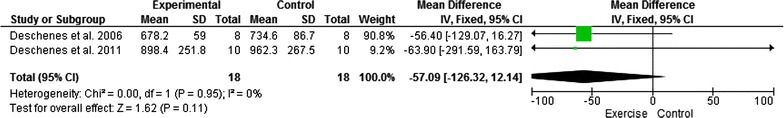
Forest plot presented information about exercise effects on fast-twitch end plate total area

Testing for overall effect did not show endurance training inducing change (p = 0.11). However, coefficient interval showed wide range of possible effect [95 % CI, −57.09 µm^2^ (−126.32, 12.14)]. Data and forest are presented in Fig. 17.

#### Stained area

**Fig. 18:**
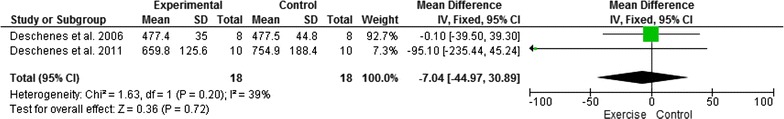
Forest plot presented information about exercise effects on fast-twitch end plate stained area

Forest plot demonstrated moderate level of heterogeneity (p = 0.20, I^2^ = 39 %) between studies. Coefficient interval showed high range interval [95 % CI, −7.04 µm^2^ (−44.97, 30.89)]. Data are showed in Fig. 18.

### Dispersion

**Fig. 19:**
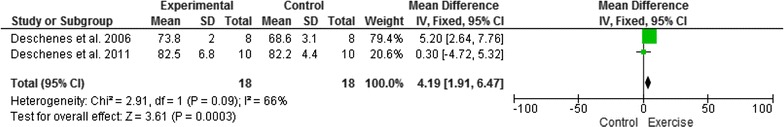
Forest plot presented information about exercise effects on fast twitch end plate dispersion

Data demonstrated moderate to high level of heterogeneity level (p = 0.09, I^2^ = 66 %) between studies. Test for overall effect showed that endurance training increased endplate dispersion by 4.19 % [95 % CI (1.91, 6.47)]. Data and forest plot are shown in Fig. 19.

### Pre-to post-synaptic coupling

**Fig. 20:**
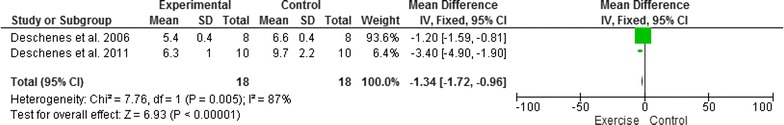
Forest plot presented information about exercise effect on fast twitch end plate pre- to post-synaptic coupling

Forest plot demonstrated high level of heterogeneity (p = 0.005, I^2^ = 87 %) between studies. Endurance training appeared to decrease pre- to post-synaptic coupling [p < 0.00001, 95 % CI, −1.34 (−1.72, −0.96)]. Data and forest plot are shown in Fig. 20.

## Discussion

Systematic reviews are articles of great scientific relevance because summarize and discuss methodological characteristics and results in a time judiciously point.

Searching for articles of great impact and scientific relevance about neuromuscular junction, we crossed keywords in some of the leading scientific literature database. Our task resulted in nine articles selected for systematic review and two for meta-analysis (young/adult NMJs). This small number of included articles is partly because of the inclusion and exclusion criteria of scientific studies. From this moment, we discussed the results found in this study. At the end of the discussion section, we presented briefly some of the cellular and molecular mechanisms that are involved in adaptations of the NMJ to exercise.

### Young and adult NMJ

#### Pre-synaptic compartment

Majority of articles included in the systematic review showed that endurance training clearly caused hypertrophy of the pre-synaptic component. According to Deschenes et al. [18], myofiber profile changes (such as myofiber increase) were unrelated to NMJ size. Among the various results that have been reported here, we could quote increased area, total area, length of the branches and branching complexity of the soleus muscle. Soleus muscle is described as primarily oxidative and has postural characteristics and features [23, 24]. Recently, Deschenes and colleagues [18] described important changes such as increased number and length of terminal branches of NMJs in type I muscle fibers of soleus. Thus, it is clear the inclusion of this muscle type in studies that aim to research physical exercise effect as favorite intervention approach. On the other hand, resistance training seemed not to induce the same adjustments to pre-synaptic compartment of soleus muscle, as did endurance training. Possibly, this issue might be explain by the difference in functional action of soleus during endurance and resistance tasks.

Meta-analysis confirmed the majority of results obtained in the systematic review. However, statistical analysis showed high level of heterogeneity in some of the analyzed parameters (total and average branch length and branch complexity). Due to the low number of included articles, we were unable to perform a sensitivity analysis. This feature would enable us to try to find the complicating factor and probable cause of this high heterogeneity. Reasoning, we can indicate that despite being young adult animals, the study by Deschenes et al. [17] used animals at 7 weeks of age. According Maltin et al. [25], animals take about 17 weeks to establish the neuromuscular pattern of muscle fiber types. Thus, we hypothesize influence of the age of the animals in the results showed by the author. Other features such as training volume, intensity and frequency might also cause divergence and we indicate a better control of these variables in future researches.

In many other muscles, of great mobilization during dynamic movement, were changed also by training. Andonian and Fahim [21] and Fahim [22] demonstrated increases on area of the nerve ending of EDL and Gluteus Maximus, respectively. However, Deschenes, Roby and Glass [18] indicated a reduction of plantaris muscle branching complexity in the trained group compared with control group. Meanwhile, another study reported that resistance training was unable to change any pre-synaptic component of that skeletal muscle [19].

For last, soleus and plantaris fast-twitch NMJs appeared not to be affect by endurance or resistance training [18, 19]. However, these studies were not corroborated by meta-analysis. This procedure showed that endurance training induced increases at all pre-synaptic components, despite moderate heterogeneity found. In addition, difference on recruitment pattern during different exercise training might also explain the difference between specific NMJ adaptations found here.

### Post-synaptic compartment

Corroborating data mentioned above, the post-synaptic compartment might also be change when faced to endurance training. Structural changes such as increased endplate area and total perimeter of the soleus muscle are reported here. On the other hand, there seems to be a difference in adapting the NMJs of fast and slow muscle fibers of the soleus and plantaris. According to Deschenes and colleagues [18], the slow fibers of the soleus and plantaris feature inversely proportional adjustments. The author demonstrated increases in area and total perimeter of the soleus endplate, while these same parameters presented inversely reduced in plantaris. In addition, the type of training also appeared to influence NMJ morphological adaptations. Endurance training induced changes in NMJs of slow fibers of both skeletal muscles. However, meta-analysis refuted this information. Forest plot showed that endurance training did not significantly affect the post-synaptic compartment of slow fibers NMJs, while many changes were found in fast-twitch fibers. Analysis of overall effect showed increased total perimeter and dispersion of the endplate and reduced stained perimeter and pre- to post-synaptic coupling of fast-twitch fibers. Yet, as previously mentioned, the age of the animal at the beginning of training period possibly affected results and could explain the adaptive morphological responses to endurance training.

On the other hand, resistance training increased endplate dispersion of the soleus but not plantaris. Finally, only resistance training induced increase in total area of the NMJs of fast-twitch myofibers. This fact is intriguing since plantaris muscle is known as a more recruited muscle during resistance exercise, and thus, possibly, more required during these tasks.

### Aged NMJ

Aging is inherent in all living beings. With advancing age, changes in various physiological systems happen and might reduce the individual’s functional capacity [4]. For the neuromuscular system, this fact is also true [26]. The aging process leads to a different adaptation of the NMJs [11, 27]. NMJ adaptation is important to compensate the constant process of denervation and reinnervation. In older animals, as well as humans, the denervation process protrudes reinnervation, causing a steady loss of motor units and consequently future physical dependence. Thus, it is important to intervene and stop the functional denervation process caused by aging.

Among the various strategies found in the scientific literature, exercise and/or physical training appeared as activity of easy implementation and low cost. Cheng et al. [11.] demonstrated that voluntary exercise training through life delayed death expectancy and presented a more compact NMJ structure at late ages.

Our systematic review included four articles, three of the effect of endurance training and only an article investigated the effect of resistance training. As previously mentioned, it was not possible to do meta-analysis of this subject.

### Pre-synaptic compartment

Clearly, endurance training affected the morphological adaptations of NMJs from soleus muscle and to a lesser extent gluteus maximus, plantaris and EDL [18, 20, 22]. Adaptations appeared to be different in NMJs of fast and slow fibers. Deschenes et al. [17] showed a reduction of the average length of the nerve terminal branches of NMJs in slow fibers and increase number of NMJ branches in fast-twitch fibers of the soleus muscle. In plantaris muscle, this adaptation seemed to happen only in NMJs of slow-twitch fibers. Still, adjustments must also be dependent on type of training, as only endurance training caused adjustments on NMJ.

### Post-synaptic compartment

The post-synaptic compartment seemed to show similar changes to pre-synaptic, but more dependent on the type of training. Deschenes and colleagues [18] showed that endurance training reduced the perimeter of stained NMJs of slow-twitch fibers of the soleus muscle, while was not identified morphological adaptation to resistance training. Moreover, NMJs from fast-twitch fibers presented increases in endplate total area and dispersion only at resistance trained. According to Deschenes et al. [19], this fact could be explain by expansions of post-synaptic Endplate structure without nerve terminal branch length increases. On the other hand, plantaris muscle only increased the pre- to post-synaptic coupling of NMJs from slow-twitch fibers during endurance training [18]. Again, it is interesting to note that plantaris muscle did not suffer any adjustments compared to soleus during resistance training [19]. This fact might be explain by volume and intensity of resistance training. Neither article included in this study did load testing sessions. There is clear the difficulty to mimic resistance training in rodent models [28, 29]. Deschenes et al. [16] mentioned that neither soleus nor plantaris suffered muscular hypertrophy. A plausible explanation is the insufficiency to achieve high load training during training sessions. Today, many research centers question the absence of methodological control of training loads and testing sessions during muscle hypertrophy experiments [28].

### Effect of exercise training on the NMJ molecular pathways

The benefits of physical exercise for humans are known for many years. However, the molecular mechanisms that coordinate the improvement of system functions are not completely known. Described above, the results presented by studies selected different adaptations in the NMJ of young and aged animals, when faced with exercise training. Possibly adaptations that occur in this structure might also be different during the course of aging.

Physical training can interfere positively in the upregulation and protein expression of several molecules and growth factors. Physical exercise can alter the expression of *Glial cell line*-*derived neurotrophic factor* (GDNF) differently in slow and fast muscle fibers, and additionally affect peripheral motor neurons [14, 30]. Wehrwein and colleagues [30] showed that 4 weeks of walking exercise on a treadmill increased GDNF content in soleus, gastrocnemius and pectoralis major muscles, while limb immobilization generated opposite effect. Recently, Gyorkos and Spitsbergen [14] examined the effect of different training intensities (10, 20, 30 and 40 m/min) on a running wheel with and without resistance on the expression of GDNF in the NMJ from plantaris muscle and adaptations of slow and fast muscle fibers of young rats. The results showed increases of 174 and 161 % of the GDNF content and 123 and 72 % of the area of the stained endplate of the groups with and without resistance, respectively. In addition, other growth factors may be involved in the NMJ adjustments induced by physical exercise. The increased expression of *Neurotrophin 4* (NT-4) appears to be activity dependent [31]. However, facts with exercise are still missing. In addition, *Brain*-*derived neurotrophic factor* (BDNF) might be increased by exercise [10]. For last, *Insulin*-*like growth factor*-*1* (IGF-1) also might be involved as a key regulator growth factor controlled by exercise [32]. This clearly demonstrates the likely effects of growth factors on the adaptation of the NMJ and subsequent innervation and reinnervation of motor neurons.

*Calcitonin gene*-*related peptide* (CGRP) acts upon a wide variety of systems. In the peripheral nervous system, CGRP increases the expression of synaptic AChR by increasing the opening time of it channels [33, 34]. Parnow et al. [35] studied the effects of endurance training (60 min/day at 30 m/min) and resistance (2 m wire mesh tower) on CGRP and AChR slow-twitch (soleus) and fast-twitch (Tibialis anterior) muscle fibers and Sciatic nerve. The results showed not to differ between muscles of different features and that both types of training increased significantly CGRP and AChR. However, it is not known the action of CGRP on hypertrophy of the NMJ, since the size of the NMJ was not measured in this study.

Physical training might still regulate many *extracellular matrix proteins* (MMP). Recently, gelatinases MMP-9 and MMP-2 were implicated in muscle adaptation to exercise. Yeghiazaryan et al. [36] found a strong gelatinolytic activity associated with Myelin sheaths within intramuscular nerve twigs. In EDL, but not Soleus, was seen an increase in the gelatinolytic activity at the post-synaptic domain of the NMJ. Author quoted that increased activity was found within punctate structures situated near the NMJ synaptic cleft. These results supported the idea that gelatinolytic activity at the NMJ might be involved in NMJ plasticity during exercise training.

We know that aging process alters the expression of several skeletal muscle proteins. However, few studies have investigated the effect of exercise on the molecular signaling of aged NMJ. Nishimune et al. [37] investigated the effects of tongue isometric exercises on active zone *protein Bassoon* of aged NMJ. The authors demonstrated that aging significantly reduced *Bassoon* levels. However, exercise stabilized and ameliorated *Bassoon* levels. Important to point out that authors used an exercise that is not voluntary. Even so, data on *Bassoon* response raised the importance of this protein in the control of calcium channels and synaptic transmission at the NMJ.

In summary, this study aimed to make a systematic review of studies investigating the effect of exercise on the compartments that form the NMJ of young, adult and aged animals. Data collected showed the opposite effect of exercise on the NMJ of young and aged animals. In young/adult animals, endurance training promoted increases on nerve terminal, total area, nerve terminal length and branching complexity of the pre-synaptic compartment. Meantime, reduced perimeter, branch numbers, terminal area, average branch lengths and branch complexity was demonstrated in the NMJ of aged animals. Similar effects have been shown on the post-synaptic compartment. In young/adult animals, increased total area and perimeter was shown. Meanwhile, aged animals demonstrated reduction of perimeter stained. Thus, physical exercise promoted hypertrophy of young/adult NMJ and compression of aged ones. Clearly, the type of exercise affects the NMJ adaptive response, since the effects appeared to be more pronounce for endurance training than resisted. Every route, methodological differences in the training protocol can directly affect the response found. Data analysis also showed that different physical training protocols and investigated muscles were used. Still, different ages used may also have affected the results and the comparison between studies. We concluded that exercise training could change differently the components of the NMJ across age.

## Methods

This systematic review was developed based on the PRISMA guideline [preferred reporting items for systematic reviews and meta-analysis] [15]. This guideline is currently use for clinical studies. Therefore, we adapted it for this research.

On December 17th, 2014, we did a systematic review search in the PubMed, Google Scholar, Science Direct, Scielo and Lilacs databases, using the following *Mesh* and *Entry terms*: [(neuromuscular junction OR junction, neuromuscular OR junctions, neuromuscular OR neuromuscular junctions OR myoneural junction OR junction, myoneural OR junctions, myoneural OR myoneural junctions OR nerve-muscle preparation OR nerve muscle preparation OR nerve-muscle preparations OR preparation, nerve-muscle OR preparations, nerve-muscle OR nerve endings OR ending, nerve OR endings, nerve OR nerve ending OR presynaptic terminals OR presynaptic terminal OR terminal, presynaptic OR terminals, presynaptic OR synaptic terminals OR synaptic terminal OR terminal, synaptic OR terminals, synaptic OR synaptic boutons OR bouton, synaptic OR boutons, synaptic OR synaptic bouton OR axon terminals OR axon terminal OR terminal, axon OR terminals, axon OR nerve endings, presynaptic OR ending, presynaptic nerve OR endings, presynaptic nerve OR nerve ending, presynaptic OR presynaptic nerve ending OR presynaptic nerve endings OR motor end-plate OR endplate, motor OR endplates, motor OR motor endplates OR motor end-plate OR end-plate, motor OR end-plates, motor OR motor end plate OR motor end-plates OR postsynaptic sites OR receptors, acetylcholine OR cholinoceptive sites OR sites, cholinoceptive OR receptors, ach OR cholinergic receptors OR ach receptors OR acetylcholine receptors OR acetylcholine OR cholinoceptors OR 2-(acetyloxy)-*n*, *n*,*n*-trimethylethanaminium OR acetylcholine l-tartrate OR acetylcholine l tartrate OR l-tartrate, acetylcholine OR acetylcholine perchlorate OR perchlorate, acetylcholine OR acetylcholine picrate OR acetylcholine picrate OR acetylcholine hydroxide OR hydroxide, acetylcholine OR acetylcholine bromide OR bromide, acetylcholine OR bromoacetylcholine OR acetylcholine chloride OR chloroacetylcholine OR acetylcholine fluoride OR fluoride, acetylcholine OR acetylcholine iodide OR iodide, acetylcholine OR acetylcholine sulfate OR acetylcholinesterase OR acetylcholine hydrolase OR hydrolase, acetylcholine OR acetylthiocholinesterase)] AND (endurance exercise OR endurance training OR exercises OR exercise physical OR exercises physical OR physical exercise OR physical exercises OR exercise isometric OR exercises isometric OR isometric exercises OR isometric exercise OR exercise aerobic OR aerobic exercises OR exercises aerobic OR aerobic exercise OR exertion OR physical fitness OR exercise therapy OR physical endurance OR physical exertion OR exertion physical OR exertions physical OR physical exertion OR physical effort OR effort physical OR efforts physical OR physical efforts OR resistance exercise OR resistive training OR resistive exercise OR resistance training OR training resistance OR strength training OR training, strength OR weight lifting strengthening programs strengthening program, weight-lifting OR strengthening programs, weight-lifting OR weight lifting strengthening program OR weight-lifting strengthening programs OR weight-lifting exercise program OR exercise program, weight-lifting OR exercise programs, weight-lifting OR weight lifting exercise program OR weight-lifting exercise programs OR weight-bearing strengthening program OR strengthening program, weight-bearing OR strengthening programs, weight-bearing OR weight bearing strengthening program OR weight-bearing strengthening programs OR weight-bearing exercise program OR exercise program, weight-bearing OR exercise programs, weight-bearing OR weight bearing exercise program OR weight bearing exercise programs OR running OR swimming OR motor activity OR activities, motor OR activity, motor OR motor activities OR physical activity OR activities, physical OR activity, physical OR physical activities OR locomotor activity OR activities, locomotor OR activity, locomotor OR locomotor activities OR voluntary exercise OR plyometric exercise OR exercise, plyometric OR exercises, plyometric OR plyometric exercises OR plyometric training OR plyometric trainings OR training, plyometric OR trainings, plyometric OR stretch–shortening exercise OR exercise, stretch–shortening OR exercises, stretch–shortening OR stretch shortening exercise OR stretch–shortening exercises OR stretch–shortening cycle exercise OR cycle exercise, stretch–shortening OR cycle exercises, stretch–shortening OR exercise, stretch–shortening cycle OR exercises, stretch–shortening cycle OR stretch shortening cycle exercise OR stretch–shortening cycle exercises). In addition, and to reinforce the search for new evidence, we inputted the following *MeSH* and *entry* related terms to the search: aging OR ageing.

On previous occasions, we were asked to include a topic that discusses some probable cellular and molecular mechanisms that interfere or induce adaptations on NMJ by exercise. Thus, we sought in the literature for specific studies and presented them at the end of the “Discussion” section. For these, we did not use any specific *Mesh* or *Entry terms*, but traditional keywords such as *IGF*-*1*, *BDNF*, *GDNF*, *Bassoon*, *MMP*, *NT*-*4* and others.

### Studies selection

#### Inclusion and exclusion criteria

We searched for animal experimental design studies that studied the exercise training effect on the NMJs components and the effect of aging on it. The inclusion criteria was articles with health young, adult or aged rodent animals, voluntary exercise intervention used (endurance, resistance, swimming or other), and detailed data on NMJ morphology. We excluded all papers that investigated exercise effect on the genetic modified animals, interventions such as surgery, muscle unloading, electrical shock stimulation, tongue exercise or the use of any drug or alimentary supplement. Rodent strain was not stated as an inclusion criteria.

### Outcomes

Outcomes of interest were nerve terminal components (branch number, total branch length, average branch length and branching complexity) and post synaptic components (total perimeter, stained perimeter, total area, stained area, dispersion and pre- to post-synaptic coupling) of slow and fast-twitch muscle fibers. We also included papers that investigate outcomes such as: endplate acetylcholinesterase and/or cholinesterase and any other NMJ morphological components.

### Extraction and analysis of data quality

We extracted data about animals strain, age, gender, exercise training parameters, primary outcomes, analyzed muscles, and primary results. Usually, clinical systematic review researches apply a data quality analysis on paper methodology through specific topic questionnaires. However, questionnaires for these proposes do not exist for animal studies. Therefore, we did not apply any data quality analysis in this study. Data as strain and/or age of rodent were not used in the search strategy not to limit the amount of included studies.

### Data synthesis and analysis

Systematic review data was organized in Tables 1, 2 and 3. For meta-analysis, Review manager software 5.3 was used to calculate heterogeneity by the I^2^, Chi^2^ and Tau^2^ values. We used I^2^ to assess heterogeneity between trials, using fixed effect models where there was high heterogeneity. We also used inverse variance method and 95 % total confidence interval.

This work is an analysis of published data, which did not require ethics committee approval.

## Abbreviations

NMJ: neuromuscular junction
AChR: acetylcholine receptor
EDL: extensor digitorum longus
IGF-1: insulin like growth factor-1
CGRP: calcitonin gene-related peptide
GDNF: glial cell line-derived neurotrophic factor
NT-4: neurotrophin 4
BDNF: brain-derived neurotrophic factor

## References

[1] Wilson MH, Deschenes MR (2005). The neuromuscular junction: anatomical features and adaptations to various forms of increased, or decreased neuromuscular activity. Intern J Neuroscience.

[2] Deschenes MR, Hurst TE, Ramser AE, Sherman EG (2013). Pre- to post-synaptic relationships of the neuromuscular junction are held constant across age and muscle fiber type. Dev Neurobiol.

[3] Ma J, Smith BP, Smith TL, Walker FO, Rosencrance EV, Koman A (2002). Juvenile and adult rat neuromuscular junctions: density, distribution, and morphology. Muscle Nerve.

[4] Gault ML, Willems ME (2013). Aging, functional capacity and eccentric exercise training. Aging Dis.

[5] Kang H, Lichtman JW (2013). Motor axon regeneration and muscle reinnervation in young adult and aged animals. J Neurosci.

[6] Shi Y, Ivannikov MV, Walsh ME, Liu Y, Zhang Y, Jaramillo CA, Macleod GT, Van Remmen H (2014). The lack of CuZnSOD leads to impaired neurotransmitter release, neuromuscular junction destabilization and reduced muscle strength in mice. PLoS One.

[7] Tamaki T, Hirata M, Uchiyama Y (2014). Qualitative alteration of peripheral motor system begins prior to appearance of typical sarcopenia syndrome in middle-aged rats. Front Aging Neurosci.

[8] Sanchis-Gomar F, Pareja-Galeano H, Mayero S, Perez-Quilis C, Lucia A (2014). New molecular targets and lifestyle interventions to delay aging sarcopenia. Front Aging Neurosci.

[9] Montero-Fernández N, Serra-Rexach JA (2013). Role of exercise on sarcopenia in the elderly. Eur J Phys Rehabil Med.

[10] Nishimune H, Stanford JA, Mori Y (2014). Role of exercise in maintaining the integrity of the neuromuscular junction. Muscle Nerve.

[11] Cheng A, Morsch M, Murata Y, Ghazanfari N, Reddel SW, Phillips WD (2013). Sequence of age-associated changes to the mouse neuromuscular junction and protective effects of voluntary exercise. PLoS One.

[12] Deschenes MR, Maresh CM, Crivello JF, Armstrong LE, Kraemer WJ, Covault J (1993). The effects of exercise training of different intensities on neuromuscular junction morphology. J Neurocytol.

[13] Deschenes MR, Judelson DA, Kraemer WJ, Meskaitis VJ, Volek JS, Nindl BC, Harman FS, Deaver DR (2000). Effects of resistance training on neuromuscular junction morphology. Muscle Nerve.

[14] Gyorkos AM, Spitsbergen JM (2014). GDNF content and NMJ morphology are altered in recruited muscles following high-speed and resistance wheel training. Physiol Rep.

[15] Liberati A, Altman DG, Tetzlaff J, Mulrow C, Gotzsche PC, Ioannidis JP (2009). The PRISMA statement for reporting systematic reviews and meta-analyses of studies that evaluate healthcare interventions: explanation and elaboration. BMJ.

[16] Crockett JL, Edergton VR, Max SR, Barnard RJ (1976). The neuromuscular junction in response to endurance training. Exp Neurol.

[17] Deschenes MR, Tenny KA, Wilson MH (2006). Increased and decreased activity elicits specific morphological adaptations of the neuromuscular junction. Neuroscience.

[18] Deschenes MR, Roby MA, Glass EK (2011). Aging influences adaptations of the neuromuscular junction to endurance training. Neuroscience.

[19] Deschenes MR, Sherman EG, Roby MA, Glass EK, Harris MB (2015). Effect of resistance training on neuromuscular junctions of young and aged muscles featuring different recruitment patterns. J Neurosci Res..

[20] Waerhaug O, Dahl HA, Kardel K (1992). Different effects of physical training on the morphology of motor nerve terminals in the rat extensor digitorum longus and soleus muscles. Anat Embryol.

[21] Andonian MH, Fahim MA (1987). Effects of endurance exercise on the morphology of mouse neuromuscular junction during ageing. J Neurocytol.

[22] Fahim MA (1997). Endurance exercise modulates neuromuscular junction of C57BL/6NNia aging mice. J Appl Physiol.

[23] Delp MD, Duan C (1996). Composition and size of type I, IIA, IID/X and IIB fibers and citrate synthase activity of rat muscle. J Appl Physiol.

[24] Roy RR, Horota WK, Kuehl M, Edgerton VR (1985). Recruitment patterns in the rat hindlimb muscle during swimming. Brain Res.

[25] Maltin CA, Delday MI, Baillie AGS, Grubb DA, Garlick PJ (1989). Fiber type composition of nine rat muscles I. Changes during the first year of life. Am J Physiol.

[26] Hopp JF (1993). Effects of age and resistance training on skeletal muscle: a review. Phys Ther.

[27] Deschenes MR, Roby MA, Eason MK, Harris MB (2010). Remodeling of the neuromuscular junction precedes sarcopenia related alterations in myofibers. Exp Gerontol.

[28] Krause Neto W, Caperuto EC, Gama EF (2013). Resistance training design for animal research: a comparative methodological study. Aust J Basic Appl Sci.

[29] Lowe DA, Alway SE (2002). Animal models for inducing muscle hypertrophy: are they relevant to clinical applications in humans?. J Orthop Sports Phys Ther.

[30] Wehrwein EA, Roskelley EM, Spitsbergen JM (2002). GDNF is regulated in an activity-dependent manner in rat skeletal muscle. Muscle Nerve.

[31] Funakoshi H, Belluardo N, Arenas E, Yamamoto Y, Casabona A, Persson H, Ibanez CF (1995). Muscle-derived neurotrophin-4 as an activity-dependent trophic signal for adult motor neurons. Science.

[32] Hameed M, Orrell RW, Cobbold M, Goldspink G, Harridge SD (2003). Expression of IGF-I splice variants in young and old human skeletal muscle after high resistance exercise. J Physiol.

[33] Fernandez HL, Ross GS, Nadelhaft I (1999). Neurogenic calcitonin gene-related peptide: a neurotrophic factor in the maintenance of acetylcholinesterase molecular forms in adult skeletal muscles. Brain Res.

[34] Leveritt M, Abernethy PJ, Barry BK, Logan PA (1999). Concurrent strength and endurance training: a review. Sports Med.

[35] Parnow A, Gharakhanlou R, Gorginkaraji Z, Rajabi S, Eslami R, Hedayati M, Mahdian R (2012). Effects of endurance and resistance training on calcitonin gene-related peptide and acetylcholine receptor at slow and fast twitch skeletal muscle and sciatic nerve in male Wistar rats. Int J Pept..

[36] Yeghiazaryan M, Cabaj AM, Sławińska U, Wilczyński GM (2015). The expression and function of gelatinolytic activity at the rat neuromuscular junction upon physical exercise. Histochem Cell Biol..

[37] Nishimune H, Numata T, Chen J, Aoki Y, Wang Y, Starr MP, Mori Y, Stanford JA (2012). Active zone protein Bassoon co-localizes with presynaptic calcium channel, modifies channel function, and recovers from aging related loss by exercise. PLoS One.

